# Candidate Gene Identification with SNP Marker-Based Fine Mapping of Anthracnose Resistance Gene *Co-4* in Common Bean

**DOI:** 10.1371/journal.pone.0139450

**Published:** 2015-10-02

**Authors:** Andrew J. Burt, H. Manilal William, Gregory Perry, Raja Khanal, K. Peter Pauls, James D. Kelly, Alireza Navabi

**Affiliations:** 1 Department of Plant Agriculture, University of Guelph, Guelph, Ontario, N1G 2W1, Canada; 2 Integrated Breeding Platform, Centro Internacional de Mejoramiento de Maiz y Trigo (CIMMYT), Carretera México-Veracruz, Km. 45, El Batán, Texcoco, Mexico 56237; 3 Department of Plant, Soil and Microbial Sciences, Michigan State University, East Lansing, Michigan 48824, United States of America; 4 Agriculture and Agri-Food Canada, Greenhouse and Processing Crops Research Centre, Harrow, Ontario, N0R 1G0, Canada; National Institute of Plant Genome Research, INDIA

## Abstract

Anthracnose, caused by *Colletotrichum lindemuthianum*, is an important fungal disease of common bean (*Phaseolus vulgaris*). Alleles at the *Co–4* locus confer resistance to a number of races of *C*. *lindemuthianum*. A population of 94 F_4:5_ recombinant inbred lines of a cross between resistant black bean genotype B09197 and susceptible navy bean cultivar Nautica was used to identify markers associated with resistance in bean chromosome 8 (Pv08) where *Co–4* is localized. Three SCAR markers with known linkage to *Co–4* and a panel of single nucleotide markers were used for genotyping. A refined physical region on Pv08 with significant association with anthracnose resistance identified by markers was used in BLAST searches with the genomic sequence of common bean accession G19833. Thirty two unique annotated candidate genes were identified that spanned a physical region of 936.46 kb. A majority of the annotated genes identified had functional similarity to leucine rich repeats/receptor like kinase domains. Three annotated genes had similarity to 1, 3-β-glucanase domains. There were sequence similarities between some of the annotated genes found in the study and the genes associated with phosphoinositide-specific phosphilipases C associated with *Co-x* and the *COK–4* loci found in previous studies. It is possible that the *Co–4* locus is structured as a group of genes with functional domains dominated by protein tyrosine kinase along with leucine rich repeats/nucleotide binding site, phosphilipases C as well as β-glucanases.

## Introduction

Common bean (*Phaseolus vulgaris* L.) with a diploid genome (2n = 2x = 22) is among the most important food legumes used for direct human consumption [1]. Among the environmental factors that affect bean production, losses caused by fungal diseases can be drastic and therefore have high importance. Although there are a number of important biotic stresses in common bean, anthracnose, caused by the hemibiotrophic fungus *Colletotrichum lindemuthianum* is one of the globally important fungal pathogens affecting sustainability of production [2]. The disease is widespread in many bean growing areas but is more prevalent in subtropical and temperate regions and can be transmitted through infected seed [3]. Cooler temperature with high humidity favors disease development that can result in complete crop failure [4]. With the adoption of no-tillage or limited tillage faming systems that increase the amount of debris hosting the pathogen to remain in the field, it is likely that anthracnose will gain more importance in the future.

In addition to using cultural practices that limit the spread of the pathogen, including the use of disease free seed, development of cultivars with resistance to different races of *C*. *lindemuthianum* is important. Developing cultivars with adequate levels of resistance is considered a viable and useful disease management strategy which results in reduced application of chemical fungicides by the farming community, thereby lowering the associated environmental risk as well as operational costs. Among the resistance mechanisms, major or race-specific resistance gene function is likely to be involved in host-pathogen recognition mechanisms triggering hypersensitive defense response, when challenged by races of the pathogen possessing corresponding avirulence alleles [5]. This mode of resistance can easily be overcome by point mutations taking place in the genetic architecture of the pathogen. Therefore, it is advantageous to deploy cultivars with multiple effective resistance genes because; multiple mutations in the pathogen would be needed to occur simultaneously in order to overcome such resistance, which is expected to be extremely rare [6]. Another approach of long lasting resistance is to develop cultivars with durable, broad spectrum resistance genes.

Preliminary attempts to understand durable or partial resistance to *C*. *lindemuthianum* in common bean have been made [7, 8]. Linkage mapping with resistance gene analogs, derived from different R gene motifs, has detected several loci associated with resistance to anthracnose as well as angular leaf spot and bean golden mosaic virus, with some loci associating with resistance to one specific disease and some other loci detecting resistance to multiple diseases [9]. Gene clusters conferring resistance to anthracnose in common bean have been recently reviewed by Ferreira et al. [2]. Similar observations of loci containing resistance genes or gene complexes conferring resistance to multiple biotic stresses have been reported in other crops such as wheat and maize [10, 11].

Anthracnose resistance genes have been designated with the abbreviation ‘Co’ followed by a number for a specific locus [2, 12]. A wide range of *C*. *lindemuthianum* races with varying virulence levels have been reported in different geographies [13]. The highly virulent fungal isolate Race 73, first reported in the state of Michigan [14], was subsequently detected in North Dakota [15], the largest bean producing state in the US, as well as in Canadian provinces of Ontario [16] and Manitoba [17]. Race 73 causes anthracnose in a significant number of commercial bean cultivars in North Dakota [18] and elsewhere in North America.

Genetic mapping and candidate gene analysis have established genetic map positions for the resistance genes among 7 of the 11 linkage groups [19, 20]. Although a majority of these race specific resistance factors exist as single genes, several are known to possess multiple allelic forms [2, 12]. Twenty genes that confer resistance to anthracnose have been described [2].

Four broadly distinct classes of resistance genes involved in defense response have been identified in different crop species. These genes are known to encode a) serine threonine protein kinase (STK) [21], b) transmembrane receptors with extracellular leucine-rich repeats (LRR) domains [22], c) a kinase group known as receptor like kinase proteins (RLK) [23] and d) proteins with a nucleotide binding site (NBS) and a LRR chain [24]. The upstream NBS domain in the fourth group can either be a region containing coiled coils (CC) or Toll/Interleukin–1 receptor-like region [25].

Many of the resistance genes encode proteins that either activate plant reaction to pathogens or have a sensory role in detecting the pathogen [26]. A majority of the cloned genes that confer race-specific disease resistance encode proteins that contain a central nucleotide-binding domain and a C-terminal leucine-rich repeat (NBS-LRR) proteins [6,27,28]. These NBS-LRR complexes are often organized into tightly linked gene clusters in plant genomes [29, 30]. As in other crop species, resistance to *C*. *lindemuthianum* in common bean is often organized as gene clusters containing independent loci as well as multiple alleles at the same locus [2, 12]. There are multiple examples of R gene clusters composed of subclasses of NBS-LRR domains in common bean genome against *C*. *lindemuthianum*. These include a R gene cluster on *Co–1* complex on the terminal end of Pv01 [12], the Co–3 complex on the short arm of Pv04, a cluster of R genes adjacent to *Co–2*, located on the terminal end of Pv11 [31], and additional clusters on Pv01, Pv04 and Pv08 [32, 33]. The resistance gene *Co-x* that confers resistance against *C*. *lindemuthianum* strain 100 has also been mapped to a gene cluster at the end of Pv01, which may be different than the *Co–1* gene complex [20]. Such complex loci have also been shown to be present in species such as rice [34], wheat [35], and barley [36].

The gene *Co–4* has been mapped to Pv08 [37] and is known to be multi-allelic [38]. Molecular markers specific to different alleles have been developed [2]. The *Co–4* locus is considered valuable since different alleles at this locus are known to control 97% of the known races of *C*. *lindemuthianum* [37]. The *Co–4* ^*2*^ allele has been reported as having broad based resistance against a range of *C*. *lindemuthianum* races [13, 39]. The SCAR markers SAS13, SH18 and SBB14 with tight linkage to *Co–4* ^*2*^ have been described [38, 40, 41]. The genomic region containing *Co–4* with high degree of similarity to tomato *Pto* gene has been further characterized and known to contain an open reading frame coding for a serine-threonine kinase [33, 37]. Melotto and Kelly [42] identified *COK–4*, a 1110 bp open reading frame as part of *Co–4* locus. A recent study by Oblessuc et al. [33] identified 18 copies of *COK–4* in addition to other predicted transcripts associated with *Co–4* locus and estimated its boundaries in a 325 Kbp region on Pv08.

Another valuable resistance gene of Andean origin, *Co-x*, has recently been characterized. Candidate gene analysis and fine mapping in the *Co-x* region using the genome sequence of G19833, the interval containing *Co-x* was refined to a 58 kb region with 8 predicted genes within the defined region on Pv01 [20]. Three of the predicted genes belonged to the Phosphoinositide specific phospholipase C (PI-PLC) family in *P*. *vulgaris*. Protein sequence analysis of the three PI-PLC candidates against the common bean G19833 genome sequence revealed the genomic location of the PI-PLC family on the distal region of Pv08 in addition to the defined 58 kb region on Pv01 [20].

Molecular markers offer significant potential in breeding for anthracnose resistance since if tightly linked markers are available, they can be used to deploy multiple resistance genes in cultivars making their breakdown due to emergence of new races considerably less probable. The presence of multiple effective genes and alleles with tightly linked markers give breeders a unique opportunity to deploy complementary gene combinations and to choose the most effective combinations in breeding strategies.

The public availability of genomic sequences greatly facilitates fine mapping and marker identification with tight linkages to the genes of interest. The common bean genome from an Andean landrace G19833 aka Chaucha Chuga has been sequenced and annotated recently (www.phytozome.org; [43]). With fine mapping capabilities using the available sequence information in common bean, it is possible that SNP markers tightly linked to known resistance loci can be used in breeding to develop cultivars with enhanced levels of resistance to *C*. *lindemuthianum*.

The objective of this study was to use a population of a cross between a susceptible and a resistant common bean cultivar that segregate for *Co–4* ^*2*^ gene to map the region with SNP markers and to identify candidate genes likely to be associated with the *Co–4* region using the genome sequence information available with the reference genome of G19833.

## Materials and Methods

### Plant materials

Ninety four F_4:5_ (F_4_ derived F_5_) recombinant inbred lines (RILs), developed using single-seed descent from a cross between anthracnose susceptible navy bean Nautica and the resistant black bean B09197, was used in the study. Nautica is a navy bean variety registered in 2007 [44], whereas B09197 is a black bean line developed at the Michigan State University, known to carry the *Co–4* ^*2*^ allele for resistance to anthracnose.

### Phenotypic evaluation

Ninety four F_4:5_ RILs and four anthracnose differential cultivars were evaluated in 2013 in a growth room facility at the Greenhouse and Processing Crops Research Centre, Agriculture and Agri-Food Canada, Harrow, Ontario, Canada. Each experimental unit was a single pot containing 3 plants. Parents and a set of control lines were also included in screening. Inoculations were conducted 10 days after emergence as the primary leaves were emerging. Inoculum was collected from fresh sporulating cultures of a characterized race 73 (binary system designation, [45] strain of *C*. *lindemuthianum* grown on modified Mathur’s agar at 25°C for 10–12 days. Spores were harvested by washing the culture plates with distilled water and filtering the extract. Inoculum solution was made by adjusting the spore concentration to 1.2 x 10^6^ per ml using a hemocytometer. Inoculum was sprayed on the unifoliate leaves until run-off was evident and inoculated plants were placed in a chamber with high humidity (> 95%) for 48 h at 22–25°C. Disease reaction was evaluated at 7 days after inoculation using a 1–9 scale [46]. Entries with ratings of 1–3 were considered resistant, and ratings 4–9 were considered susceptible. The control lines used were Michelite (binary system designation: 1) Perry Marrow (4), Cornell 49242 (8), Widusa (16), and Mexico 222 (64). The expected reaction of race 73 on these cultivars is a strong susceptible response on Michelite, Cornell 29242 and Mexico 222, and a resistant or incompatible reaction on Perry Marrow and Widusa.

### Genotyping

Leaf tissue samples were harvested from young leaves of multiple plants of each RIL and the parents. Young leaf samples (100 mg) were frozen in liquid nitrogen and ground using an AutoGrinder 48 (AutoGen Inc., Holliston, MA, USA). After incubation with plant lysis buffer (AutoGen AG00121) at 65°C for 30 min, DNA was automatically extracted using an AutoGen 850 alpha DNA automatic system following the manufacturer’s protocol (AutoGen Inc.).

### SCAR marker screening

Previously identified anthracnose resistance molecular markers, polymorphic between Nautica and B09197, were used to genotype the RIL population. The SCAR markers used with linkage to *Co–4* were SAS13 [38], SH18 and SBB14 [47]. PCR amplifications were performed in 25 μL reaction volumes with 1 μL genomic DNA (25 ng), 0.4 units of GOTaq DNA polymerase (Promega), 2.5 μL 10 X polymerase buffer, 200 mM dNTP, and 1 mM MgCl_2_ and 0.15mM each primer. Amplification conditions were 3 min at 94°C followed by 35 cycles of 10 s at 94°C, 30 s at 60°C and 1 min at 72°C. The PCR amplifications were performed in a Master Cycler nexus GSx1 (Eppendorf) using clear unskirted 96-well MultiplateTM PCR plate (BIO-RAD). The PCR products were separated with the Qiaxcel advanced capillary (QIAGEN) system using either QIAxcel DNA High Resolution Kit or QIAxcel DNA Fast Analysis Kit according to the manufacturer’s protocol.

### SNP genotyping

The population was also genotyped with a panel of SNP markers using the Illumina Infinium platform, part of which was developed using two next generation sequencing methods with a multi-tier reduced representation library [48]. Genotyping was performed at the Genome Quebec Innovation Center (Montreal, QC, Canada) using the Sequenom iPLEX Gold Assay (Sequenom, Cambridge, MA, USA). The SNP panel with 5361 markers were used to genotype the parents and 94 F_4:5_ RILs of the population. Polymorphisms were observed for 558 SNP markers.

### Sequence annotation

SNP markers with significant association with the phenotypic data along with the SCAR markers linked to *Co-*4 were assembled into a linkage group using MapChart 2.2 [49]. The marker trait association and QTL analysis was performed using WinQTL cartographer 2.5 [50]. The SNP markers highly associated with anthracnose resistance in Nautica x B09197 population were located in silico in G19833 (http://www.phytozome.org; [51]). The derived nucleotide sequences of the interval identified by the most significant SNP markers were used as a query against the sequence of Pv08 for the identification of potential annotated genes associated with disease resistance using local BLAST analysis using the CLC Genomics Workbench built-in BLAST functionality (CLC bio, QIAGEN Co.). The identified annotated *P*. *vulgaris* genes were used in BLAST analysis of GenBank non redundant database in order to identify candidate genes associated with disease resistance in other plant spp. [52]. Once annotated candidate genes putatively associated with disease resistance were identified, these sequences were aligned with each other at nucleotide and amino acid level to study the relationships of the candidate genes and for the identification of distinct groups based on nucleotide and amino acid sequence similarity.

## Results and Discussion

**Fig 1:**
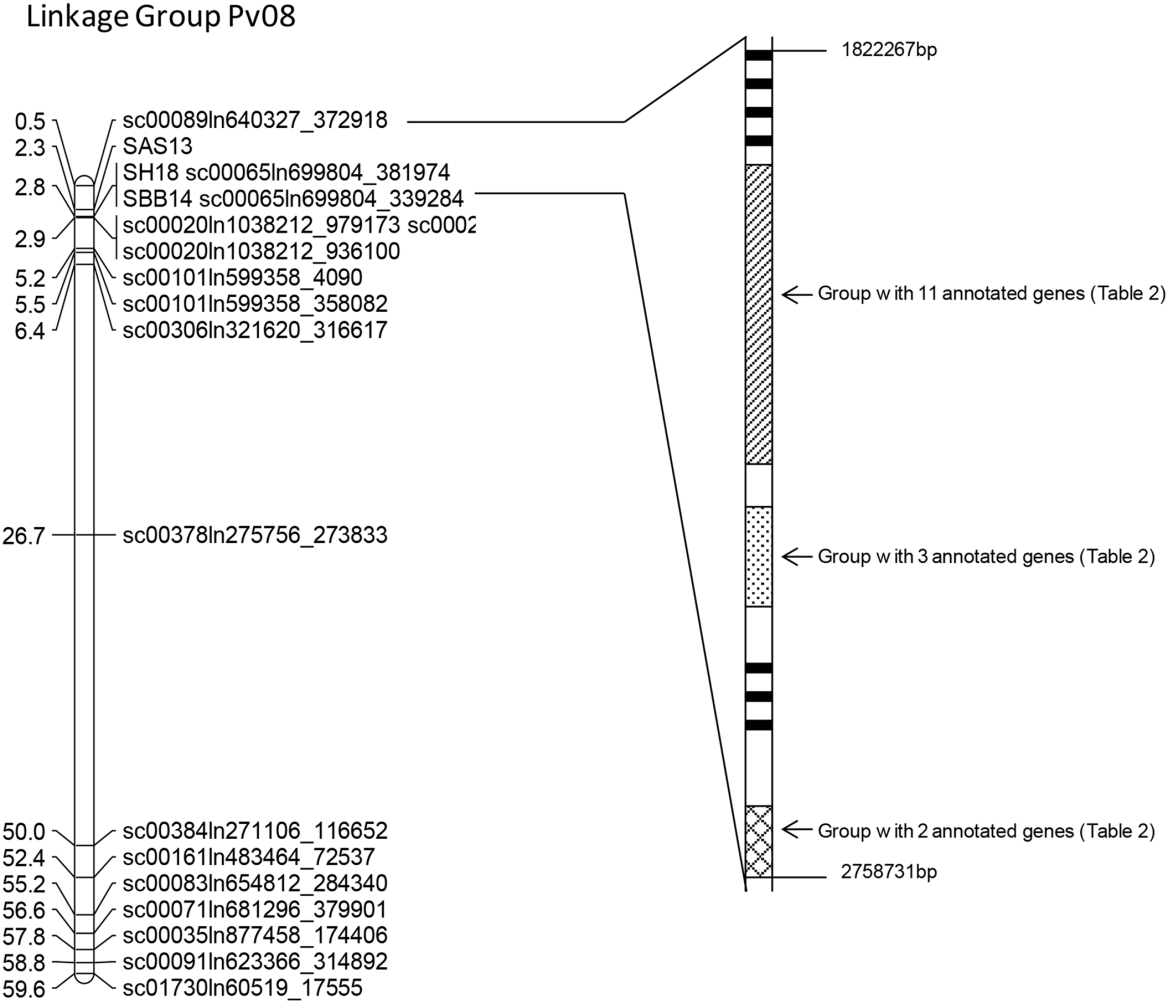
*Phaseolus vulgaris* chromosome Pv08 linkage group associated with the SCAR and SNP markers. The solid lines originating from the linkage map indicate the physical region selected for candidate gene search. The solid bars indicate the annotated genes with no similarity to other members of the annotated genes. The different shaded regions indicate the groups of annotated genes with high degree of nucleotide similarity.

Anthracnose disease ratings for *C*. *lindemuthianum* Race 73 of the 94 F_5_ families of Nautica x B09197 showed a distribution pattern with more entries falling in the resistant category. It is possible that the population showed distorted segregation towards resistance or inadvertent selection for resistance has taken place in early generations. The distribution pattern of the anthracnose resistance rating among the entries used is included in S1 Fig. Out of the 5361 markers used for genotyping, only 538 showed polymorphisms and were used for mapping in the 94 F_4:5_ RIL population. The 10.4% level of polymorphism observed was low. The two parents of the mapping population belonged to navy and black bean market classes, both of which are of Mesoamerican in origin. A recent QTL mapping study by Yuste-Lisbona et al [53] with 3700 AFLP, 1035 SSR and 251 SNP markers revealed 7.5%, 10.2% and 7.2% polymorphisms respectively between the common bean parents used. It is possible that the level of genetic diversity among cultivated beans is relatively low. Single marker analysis and interval mapping using WinQTL cartographer with the mapping population identified a group of SNP and SCAR markers with significant association with anthracnose resistance (Table 1, S1 File). This marker group including the SCAR markers SAS13, SH18 and SBB14, known to be associated with *Co–4* ^*2*^ allele and the *Co–4* locus was assembled in a linkage group according to the physical positions of the SNP markers on Pv08. Some markers that were clustered together were removed in order to increase clarity (Table 1; Fig 1). Since *Co–4* is the only known anthracnose resistance gene located on Pv08 and is the only gene with major effects segregating for resistance to race 73 in the Nautica x B09197 population, it is likely that the identified group of SNP markers span the genomic region of the *Co–4* gene. Within a 59.1 Mb region, there were several SNP markers and SCAR markers that had highly significant association with anthracnose resistance (Table 1). Within this physical region, markers within a 2.35 Mb region, flanked by the SNP marker sc00089ln640327_372918 (497385 bp) and sc00065ln699804_339284 (2843266 bp) that had the highest association with anthracnose resistance based on the likelihood ratios as well as the larger amount of the phenotypic variation (R^2^) explained by markers (Table 1). Genomic sequences within this 2.35 Mb region were used as a query in local BLAST searches against the genomic sequences of Pv08 of G19833 (www.phytozome.org; [43]).

**Table 1:** Results of the single marker analysis with anthracnose ratings of the 94 F_4:5_, recombinant inbred lines of B09197 x Nautica populations, indicating the physical location of the markers, likelihood ratio, the levels of significance and the % of variation (R^2^) explained by the markers.

Marker #	Marker Name	Mbp^a^	LR Ratio^c^	F value	Probability	Significance	R^2^
1	sc00089ln640327_372918	0.497385^b^	22.1	24.34	0.000004	****	0.173
2	SAS13	2.349419	159.6	410.72	0	****	0.817
3	SH18	2.76164	134	290.83	0	****	0.76
4	sc00065ln699804_381974	2.801265	31	36	0	****	0.237
5	SBB14	2.810566	113.6	216.19	0	****	0.687
6	sc00065ln699804_339284	2.843266^b^	75.5	113.51	0	****	0.513
7	sc00020ln1038212_979173	2.887738	28.8	33	0	****	0.225
8	sc00020ln1038212_942609	2.922646	43.6	54.29	0	****	0.334
9	sc00020ln1038212_936100	2.929155	27.2	30.9	0	****	0.231
10	sc00101ln599358_4090	5.150618	14	14.76	0.000224	***	0.122
11	sc00101ln599358_358082	5.502606	24.8	27.73	0.000001	****	0.199
12	sc00306ln321620_316617	6.391685	11.2	11.63	0.000965	***	0.086
13	sc00378ln275756_273833	26.67345	2.2	2.2	0.141117		0.004
14	sc00384ln271106_116652	49.95888	4.1	4.08	0.046297	*	0.042
15	sc00161ln483464_72537	52.44377	9.5	9.82	0.002322	**	0.09
16	sc00083ln654812_284340	55.21913	8	8.15	0.005327	**	0.086
17	sc00071ln681296_379901	56.64482	0.3	0.29	0.592207		0.004
18	sc00035ln877458_174406	57.80865	5	5.05	0.02707	*	0.06
19	sc00091ln623366_314892	58.77255	0.2	0.23	0.630199		0.006
20	sc01730ln60519_17555	59.61381	0.2	0.23	0.635274		0.005

^a^Mega base pairs.

^b^Physical megabase pair region used for BLAST search against the chromosome Pv08 of G19833.

^c^Likelihood ratio.

*.**,***,****Significant at the 0.05, 0.01, 0.001, and 0.0001 probability levels, respectively.

### Potential Candidate Genes

Local BLAST searches using the 2.35 Mb physical nucleotide interval associated with the peak QTL region to query Pv08 sequences of G19833 identified 296 unique annotated genes. These individual annotated genes were used in BLAST nucleotide searches against the GenBank non-redundant database against all plant species in order to identify candidate genes associated with disease resistance based on nucleotide sequence similarity. A majority of queries identified hypothetical or uncharacterized proteins or predicted putative functions that are not known to be related to defense response against pathogens. There were some sequences with putative functions such as zinc finger proteins, and transcription factors. The possible function of the uncharacterized proteins and transcription factors in disease response cannot be completely ruled out. However, thirty two annotated genes identified sequences from various plant species with predicted protein domains that are known to be involved in defense response reaction against pathogens. Table 2 summarizes the 32 annotated potential candidate genes, their physical location on Pv08 and the putative predicted function resulting from Phytozome functional annotations. The functional annotations identified by Phytozome were also confirmed by BLAST searches with GenBank non-redundant database searches. The majority of the annotated genes identified sequence similarities with classical defense response genes associated with protein domains containing leucine rich repeats (LRR), receptor like protein kinases (RLK), and protein tyrosine kinase (PTK) that are established protein domains of characterized genes associated with race specific disease resistance (Table 2). Twenty seven of the 32 potential candidate genes had LRR-Kinase domains as predicted putative functional annotation. One candidate gene had a potential Phosphatidylinositol 3- and 4-kinase domain and another one had a PI-PLC domain containing predicted protein domain. Three potential candidate genes had predicted endo–1,3-β-glucanase domains. The length of the coding sequences of these potential candidate genes varied from 660–2595 bases (Table 2). The remaining 264 annotated genes and the length of the genomic DNA along with their functional annotations are given in S1 Table.

**Table 2:** Results of the putative candidate genes associated with anthracnose resistance identified by *in-silico* searching of the highly significant 2.35 Mbp region with the common bean chromosome Pv08 of G19833 genomic sequences. The annotated gene identifications, their physical locations on Pv08 along with predicted putative functions identified by protein domain search tools Panther, Pfam, KOG and or NCBI along with the lengths of the coding sequences are presented.

Annotated gene ID	Location (Phytozome)	Predicted Putative Function ^e^	Coding sequence (bp)
Phvul.008G021000	Chr08: 1822267–1827996	LRR-Receptor like protein kinase/PTK-/STK	2232
Phvul.008G022700	Chr08: 1932377–1936975	LRR/LRR-N terminal domain/PK	1872
Phvul.008G025200	Chr08: 2136230–2140612	LRR-Receptor like protein kinase/PTK-/STK	1266
Phvul.008G025300	Chr08: 2141431–2145714	PI–3 and 4-kinase/PI-4-kinase	1926
Phvul.008G026600^a^	Chr08: 2247601–2248925	LRR-Receptor like protein kinase/PTK-/STK	1038
Phvul.008G026700^a^	Chr08: 2251608–2254244	LRR-Receptor like protein kinase/PTK	660
Phvul.008G026900^a^	Chr08: 2260520–2262552	LRR-Receptor like protein kinase/PTK-/STK	1074
Phvul.008G027100^a^	Chr08: 2268740–2269922	LRR-Receptor like protein kinase/PTK-/STK	1041
Phvul.008G027200^a^	Chr08: 2272940–2274087	LRR-Receptor like protein kinase/PTK-/STK	1038
Phvul.008G027300^a^	Chr08: 2276317–2277441	LRR-Receptor like protein kinase/PTK-/STK	1026
Phvul.008G028200^a^	Chr08: 2344633–2346203	LRR-Receptor like protein kinase/PTK-/STK	1035
Phvul.008G028300^a^	Chr08: 2348905–2350034	LRR-Receptor like protein kinase/PTK-/STK	1047
Phvul.008G028400^a^	Chr08: 2355100–2356125	LRR-Receptor like protein kinase/PTK-/STK	897
Phvul.008G028500^a^	Chr08: 2359858–2360982	LRR-Receptor like protein kinase/PTK-/STK	1044
Phvul.008G028600^a^	Chr08: 2367181–2369491	LRR-Receptor like protein kinase/PTK-/STK	1128
Phvul.008G029500^a^	Chr08: 2432573–2434137	LRR-Receptor like protein kinase/PTK-/STK	1032
Phvul.008G029600^a^	Chr08: 2436788–2438053	LRR-Receptor like protein kinase/PTK-/STK	1014
Phvul.008G029700^a^	Chr08: 2440665–2441768	LRR-Receptor like protein kinase/PTK-/STK	1101
Phvul.008G029800^b^	Chr08: 2444726–2446098	LRR-Receptor like protein kinase	828
Phvul.008G029900^a^	Chr08: 2456766–2457454	LRR-Receptor like protein kinase/PTK-/STK	660
Phvul.008G030100^a^	Chr08: 2480964–2482269	LRR-Receptor like protein kinase	1032
Phvul.008G030200^b^	Chr08: 2486348–2488942	LRR-Receptor like protein kinase/PTK-/STK	2595
Phvul.008G030400^b^	Chr08: 2493400–2495916	LRR-Receptor like protein kinase/PTK-/STK	2517
Phvul.008G030700^b^	Chr08: 2508216–2510975	LRR-Receptor like protein kinase/PTK-/STK	2577
Phvul.008G030800^b^	Chr08: 2531925–2534912	LRR-Receptor like protein kinase/PTK-	2601
Phvul.008G031000^c^	Chr08: 2551112–2553533	Glycine max PI-PLC X domain-containing protein	1095
Phvul.008G031100	Chr08: 2556622–2557557	LRR-Receptor like protein kinase/PTK-/STK	936
Phvul.008G031200	Chr08: 2559062–2564009	NB-ARC domain/LRR protein/Apoptotic ATPase	2784
Phvul.008G031300^a^	Chr08: 2565165–2568318	LRR-Receptor like protein kinase/STK	1020
Phvul.008G033000^d^	Chr08: 2728468–2730923	Glycosyl hydrolase /Predicted endo–1,3-β-glucanase	2004
Phvul.008G033100^d^	Chr08: 2745218–2747268	Glycosyl hydrolase /Predicted endo–1,3-β-glucanase	1968
Phvul.008G033200^d^	Chr08: 2756087–2758731	Glycosyl hydrolase /Predicted endo–1,3-β-glucanase	1968

^a^17 annotated genes that form a cluster due to nucleotide/predicted protein similarity

^b^5 annotated genes that form a cluster due to nucleotide/predicted protein similarity

^c^Functional annotation for Phvul.008G031000 was identified in BLAST search with GenBank non redundant database search.

^d^3 annotated genes that form a cluster due to nucleotide/predicted protein similarity

^e^Predicted protein function as identified by Phytozome—Panther/Pfam/KOG and or NCBI

LRR-Leucine rich repeat; PTK—Protein Tyrosine Kinase; STK—Serine Threonine Protein Kinase; PK—protein Kinase; PI- Phosphatidylinositol

The physical chromosomal region associated with disease resistance related candidate genes identified in G19833 spanned 936.46 kb on Pv08 (Fig 1 and Table 2). The peak QTL region that had 2.35 Mb was therefore refined and narrowed down to 936.46 kb after BLAST searches with the G19833 Pv08 sequences. The G19833 is likely to carry the *Co–1* and null allele at the *Co–4* locus, although it has not been fully characterized for reaction to anthracnose. Since the physical location covers a relatively large region, it is possible that the *Co–4* locus is organized as a complex locus or there are islands of genes with similar functions associated with *Co–4* on Pv08. The functional annotations identified by phytozome indicate that a majority of potential candidate genes had domains containing LRR, RLK and PTK but there were some candidate genes with other functional domains as described before, suggesting the complexity of the locus. Oblessuc et al. [33] determined the boundaries of *Co–4* locus to a 325 Kb region close to telomere on Pv08 using genetic analysis, phylogeny and sequence data with a population of 98 F_2_ individuals segregating for *Co–4* ^*2*^ allele.

In order to study the relationship of different annotated genes putatively identified to be associated with *Co–4*, each annotated candidate gene was used as a query against the rest of the annotated candidate genes to observe nucleotide and protein similarities. We used a high stringency with the E values <E^−45^ as the cutoff value in nucleotide similarity searches. The annotated candidate genes Phvul.008G021000, Phvul.008G022700, Phvul.008G025200 and Phvul.008G025300, although considered to contain LRR and/or kinase activity, did not have any sequence similarity with other annotated candidate genes (Table 2). The annotated gene Phvul.008G031000, did not identify any functional annotation in Phytozome but during BLAST searches with GenBank non-redundant database, sequence similarity was identified to phosphoinositide-specific phospholipases (PI-PLC). No sequence similarity with any other annotated gene in our collection was identified. Richard at al. [20] identified three PI-PLC genes in association with the gene *Co-x* on Pv01 that gives broad spectrum resistance to *C*. *lindemuthianum*. The PI-PLC genes reported in association with the *Co-x* gene do not belong to the classical resistance genes containing NBS-LRR domains [20]. Common bean PI-PLC gene clusters have been located on Pv01 and Pv08 [20]. The length of the coding region of the Phvul.008G031000 candidate gene with putative PI-PLC domains we identified was 1095 nucleotides. Richard et al. [20] identified four annotated sequences on Pv08 after searching the sequence information of G19833 for sequence similarity with the candidate gene found to be in association with *Co-x* with PI-PLC domains. An analysis of nucleotide similarity between Phvul.008G031000 and Phvul.008G224200 with 1818 nucleotides, which is one of the annotated genes located on Pv08 by Richard et al. [20], identified only a low level of nucleotide similarity (data not shown). The relationship between the annotated genes identified and located on Pv08 by Richard et al. [20] and *Co–4* is unknown. It is possible that the annotated gene Phvul.008G031000 identified in this study is a distinct form that is divergent from the annotated genes that has PI-PLC domains associated with *Co-x* related resistance on Pv01. Genes divergent from *Co-x* PI-PLC domains may be involved in the resistance conferred by *Co–4*. The NCBI BLAST search based on the sequence of Phvul.008G031100 identified only one association with *Glycine max* receptor-like protein kinase and did not have any sequence similarity with the other set of annotated genes found in our study. Further scrutiny of Phvul.008G031100 is required before it can be considered as a potential disease resistance gene. Phvul.008G031200, identified as a NB-ARC/LRR containing protein, also had high stringency sequence similarities with *Glycine max* putative disease resistance proteins in BLAST searches. However, it did not have any sequence similarity with other annotated genes in our study. Phvul.008G031200 was previously identified to be a candidate gene associated with QTL for partial resistance to anthracnose on Pv08 [32]. Four of these seven annotated genes with no sequence similarity to the rest are localized adjacent to each other with 104.3 kb between Phvul.008G021000 and Phvul.008G022700 and 199 .25 kb between Phvul.008G022700 and Phvul.008G025200 followed by 819 base between Phvul.008G025200 and Phvul.008G025300. The other three annotated genes are also adjacent to one another as a separate group with 3089 bases between Phvul.008G031000 and Phvul.008G031100 and 1505 bases distance between Phvul.008G031200 and Phvul.008G031100. At least six of these seven annotated genes with the possible exception of Phvul.008G031100 appear to be unique genes with predicted protein domains that are characteristic of disease resistance genes that have no significant nucleotide similarity to other annotated genes found in the study.

**Fig 2:**
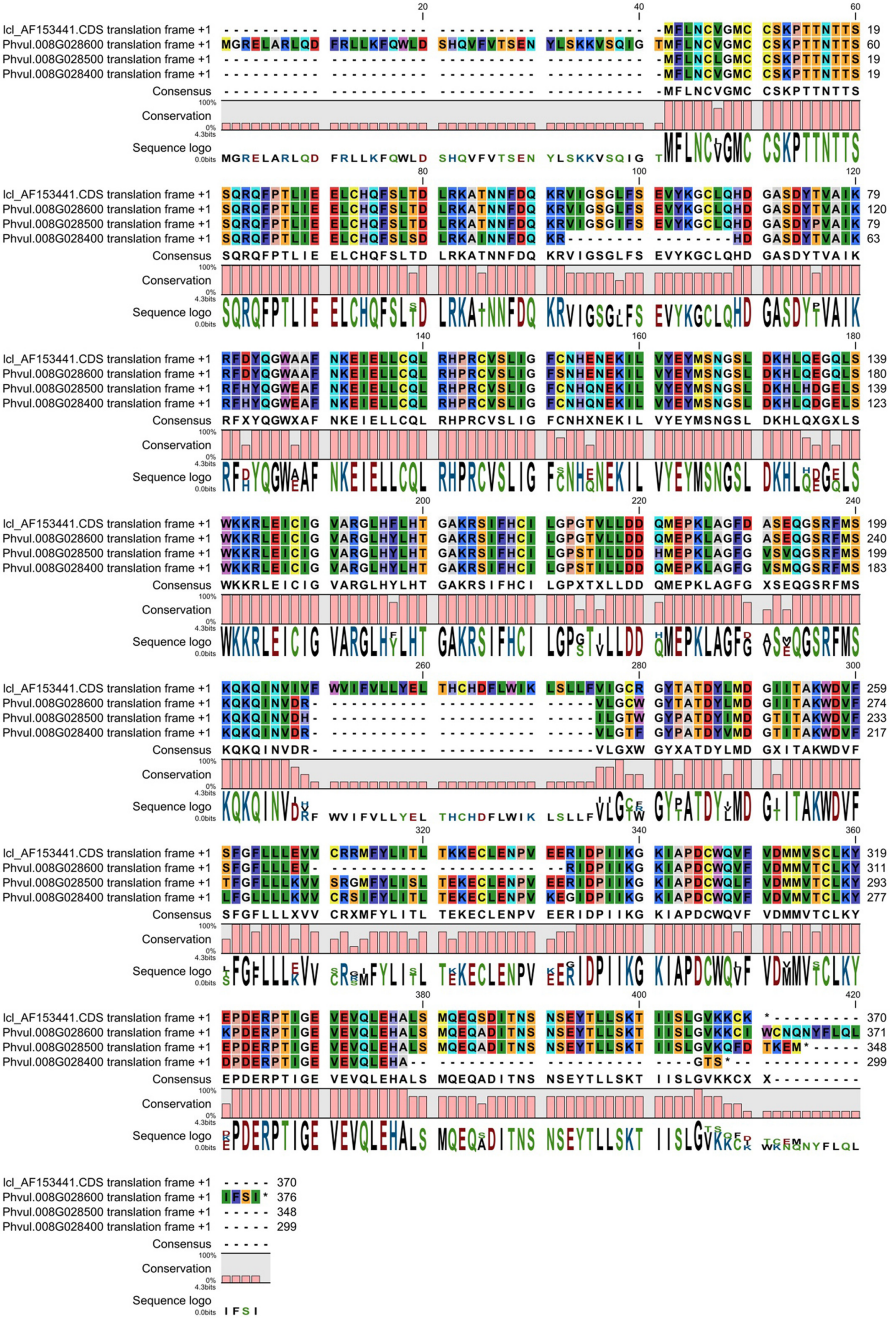
Alignment of predicted protein sequence of gene *COK–4* with the annotated genes Phvul.008G028300, Phvul.008G028400, Phvul.008G028500 and Phvul.008G028600.

In addition to the seven annotated genes that had no sequence similarity to other putative candidate genes in our study, there were 25 annotated genes that showed nucleotide, amino acid and predicted protein sequence similarities to other members of the putative candidate genes. These 25 annotated sequences belonged to three distinct groups (Fig 1 and Table 2). In one group, there were 17 sequences that had high level of similarity at nucleotide and amino acid sequences and all of them contained LRR-RLK predicted protein domains. Table 3 summarizes the nucleotide alignment details of the 17 annotated genes. The coding sequence length among this group of annotated genes ranged from 660–1128 bases (Table 3). Although there was a high degree of nucleotide sequence similarity among the 17 annotated genes, there were a number of single nucleotide polymorphisms (SNP) and insertions/deletions among the members that characterized this group (S2 Fig). Phvul.008G028600 with a coding sequence length of 1128 bp had a unique 123 bp upstream sequence. Phvul.008G029700 had a unique insertion of 63 bp and Phvul.008G026700 had a unique insertion of 9 bp. GeneBank nun-redundant database searches revealed four annotated genes in this group that identified a high level of sequence similarity to the *COK–4* candidate gene with a coding sequence length of 1110 nucleotides that is known to be in close association with *Co–4* ^*2*^ [42]. Similarity statistics of the annotated gene Phvul.008G028300 (AF 153441/GI 9796477; 95% identity, e = 0, 49 mismatches); Phvul.008G028400 (AF 153441/GI 9796477; 95% identity, e = 0, 37 mismatches); Phvul.008G028500 (AF 153441/GI 9796477; 96% identity, e = 0, 39 mismatches); Phvul.008G028600 (AF 153441/GI 9796477; 98% identity, e = 0, 18 mismatches) indicate the high degree of nucleotide sequence similarities in the coding regions between the *Co–4* candidate gene and the four genes identified in the study. The complete nucleotide alignment of the coding sequences of the four annotated genes with the *COK–4* candidate gene is available in S3 Fig. Phvul.008G028400 had a coding sequence length of 897 bp which is the shortest of the four sequences. It had a deletion of 48 bp region compared to *COK–4* and the other two annotated sequences from position 153 bp (S3 Fig). Compared to the *COK–4* sequence, the annotated genes Phvul.008G028300, Phvul.008G028400 and Phvul.008G028500 contained more than 30 SNPs whereas the fourth annotated sequence Phvul.008G028600, with a coding sequence length of 1128 nucleotides had 12 SNPs. Phvul.008G028600 also had a 69 nucleotide deletion from position 849 that was unique to this annotated gene in comparison to *COK–4* and the other three annotated genes. Phvul.008G028600 had an upstream 123 nucleotide region unique to this annotated gene. All four annotated genes had a 75–80 nucleotide deletion closer to the downstream region compared to *COK–4*, whereas the *COK–4* had a unique deletion of 73 nucleotides compared to the four annotated genes at the downstream end (S3 Fig). The physical distance between Phvul.008G028300 and Phvul.008G028400 is 5.0 kb whereas Phvul.008G028400 and Phvul.008G028500 is separated by 3.7 kb and the distance between Phvul.008G028500 and Phvul.008G028600 is 6.1 kb. Melotto and Kelly [42] established that the gene *COK–4* is part of the *Co–4* locus and has a similar function as *Pto* gene in tomato [21]. The alignment with protein sequence similarities between *COK–4* and the four annotated sequences is shown in Fig 2. The sequences contained a significant number of residues of leucine and isoleucine, typical of LRR domains. The *COK–4* candidate gene AF 153441 had an insertion of 26 amino acid residues (Fig 2). Although Phvul.008G028600 has an amino acid sequence with 41 extra residues at the upstream end and 9 extra residues at the downstream end compared to the start codon of the other three members of this group and *COK–4*, the four annotated genes had predicted amino acid similarity of over 90% with *COK–4*. Melotto et al. [37] indicated the presence of multiple copies of *COK–4* in the bean genome. A study by Oblessuc et al. [33] identified 18 *COK–4* related genes on Pv08. Interestingly, all the members of the group of 17 annotated genes that had high degree of sequence similarity in our study are among the 18 genes reported by Oblessuc et al. [33]. Therefore, in addition to the four annotated genes that we identified to be associated with *COK–4* gene reported by Melotto and Kelly [42], the other 13 genes in the cluster are also functionally related to *COK–4*. Since we used the genomic sequences flanked by the markers that gave high association with anthracnose resistance, it is likely that the region that was identified in our study covers a broader set of potential candidate genes in addition to the *COK–4* gene cluster (Table 2). The 17 annotated genes in this group spanned genomic region of 317.6 kb. This region overlapped the second group of five related annotated genes as well as three annotated genes that did not have significant sequence similarities to other members (Table 2).

**Table 3:** Details of the nucleotide alignment similarities of the 17 annotated genes that formed a group with high similarity among the 23 annotated genes identified in Table 1 to be putatively associated with anthracnose resistance.

Annotated gene ID	E-value	Score	%Gaps	Location (Phytozome)	Length (bp)	Coding sequence (bp)
Phvul.008G028600	0	774	8	Chr08: 2367181–2369491	2310	1128
Phvul.008G028400	0	710	9	Chr08: 2355100–2356125	1025	897
Phvul.008G028500	0	414	0	Chr08: 2359858–2360982	1124	1044
Phvul.008G028300	1.19E-168	354	0	Chr08: 2348905–2350034	1129	1047
Phvul.008G029700	3.00E-108	232	17	Chr08: 2440665–2441768	1103	1101
Phvul.008G026900	7.92E-50	114	12	Chr08: 2260520–2262552	2032	1074
Phvul.008G028200	7.74E-49	112	6	Chr08: 2344633–2346203	1570	1035
Phvul.008G029500	6.69E-93	201	10	Chr08: 2432573–2434137	1564	1032
Phvul.008G029600	7.74E-49	112	9	Chr08: 2436788–2438053	1265	1014
Phvul.008G029900	1.63E-81	178	13	Chr08: 2456766–2457454	688	660
Phvul.008G030100	5.22E-82	179	13	Chr08: 2480964–2482269	1305	1032
Phvul.008G027300	1.63E-81	178	8	Chr08: 2276317–2277441	1124	1026
Phvul.008G027100	1.63E-81	178	10	Chr08: 2268740–2269922	1182	1041
Phvul.008G026700	1.63E-81	178	9	Chr08: 2251608–2254244	2636	660
Phvul.008G026600	1.56E-79	174	11	Chr08: 2247601–2248925	1324	1038
Phvul.008G031300	3.10E-59	133	15	Chr08: 2565165–2568318	3153	1020
Phvul.008G027200	1.42E-75	166	14	Chr08: 2272940–2274087	1147	1038

The five annotated genes that formed a second group with high degree of nucleotide similarity also were identified during BLAST searches as having predicted protein domains containing LRR/RLK activity (Fig 1 and Table 2). The interval between Phvul.008G029800 and Phvul.008G030200 was 40.2 kb and there were two annotated genes from the group of 17 within this interval. There was a 4.4 kb interval between Phvul.008G030200 and Phvul.008G030400 and a 12.3 kb interval between Phvul.008G030700 and Phvul.008G030400. The interval between the last member of this group Phvul.008G030800 and Phvul.008G030700 was 20.9 kb (Table 2). The five sequences had a number of SNPs as well as polymorphisms caused by insertions and deletions (data not shown). The amono acid alignment of the five members is in S4 Fig. The annotated gene Phvul.008G029800, being the shortest of the group having only 828 bp coding sequence (276 amino acid residues) had a large deletion in the upstream end (Table 2, S4 Fig). It is possible that Phvul.008G029800 is a truncated gene associated with other members of this group. The insertions and deletions of amino acid residues were more common in the upstream end among the members of this group (S4 Fig). Local batch BLAST searches between the previous group and this group did not identify any significant similarity at nucleotide level. It is possible that this is a distinct group of genes with predicted proteins containing LRR-Kinase like domains that may have evolved separately from the previous group.

There were three annotated genes, Phvul.008G033000, Phvul.008G033100 and Phvul.008G033200 with 1,3/1,4 β-glucanase activity that formed a third group (Fig 1 and Table 2). Phvul.008G033000 had a coding sequence length of 2004 nucleotides whereas Phvul.008G033100 and Phvul.008G033200 had a coding sequence length of 1968 bp (Table 2). The three annotated genes in this group are adjacent to each other spanning a region of 30.3 kb. There were a considerable number of SNPs among this group of annotated genes within the coding sequences (data not shown). These polymorphisms are reflected in the amino acid alignment (S5 Fig). Phvul.008G033000 had extra nine amino acid residues at the upstream end compared to the other two members of this group (S5 Fig). The amino acid alignment was stronger between Phvul.008G033100 and Phvul.008G033200. The role of 1,3-β-glucanases in fungal disease resistance is documented in multiple studies [54, 55]. 1,3-β-glucanases are known to be involved in induced systemic resistance and age-acquired resistance in plant spp. [56, 57]. The group of annotated genes with possible 1,3-β-glucanases activity, is 160.1 kb away from the closest annotated gene containing LRR-RLK protein structures. Although there are no published reports linking 1,3-β-glucanases activity with anthracnose resistance, it is possible that genes with potential glucanase activity may play a defense role against invading fungi including *C*. *lindemuthianum*.

Based on Phytozome functional annotations it is difficult to distinctly identify different members of putative candidate genes identified in our study, except the three annotated gene candidates that have 1,3-β-glucanase activity. The majority of the candidate annotated genes identified predicted functions containing LRR-RPK and or protein tyrosine kinase domains. Twenty seven of the 32 candidates that were identified in our study had some common features in their predicted functional domain activities that involved LRR/kinase domains; the exceptions were Phvul.008G025300 that had only domains with phosphatidylinositol 3-and 4-kinase activity, Phvul.008G031000 that was associated with PI-PLC domain activity, and Phvul.008G033000, Phvul.008G033100 and Phvul.008G033200 that had predicted 1,3-β-glucanase activity. In addition to LRR domains, Phvul.008G031200 also had NB-ARC domain (Table 2). Association of NB-ARC domains in regulating disease resistance is documented [58]. As mentioned previously, not all annotated candidate genes with similar predicted functional domains identified significant similarity at the nucleotide level at the threshold levels used. It is possible that there is a significant degree of divergence among different annotated genes containing similar functional motifs. This study adds further evidence that the locus *Co–4*, which includes *COK–4*, is organized as a cluster of genes containing members that have sequence and functional similarities as well as some divergent members.

### Similarity search in *P*. *vulgaris* genomic sequence

The coding sequence regions of the 32 annotated potential candidate genes on Pv08 were used individually to search for sequence homology with the genomic sequence of G19833 using Phytozome (www.phytozome.org). A cutoff stringency E value of <E^−63^ was used to identify similar sequences in other regions of the G19833 genome (Table 4). Phvul.008G021000 with a coding sequence length of 2232 nucleotides identified a single location on Pv03 spanning 2567 nucleotides with high level of homology with domains related to LRR-STK with Kinase activity. Phvul.008G022700 with a coding region of 1872 nucleotides identified 4 specific regions located on Pv02, Pv03, Pv04 and Pv07 with similar predicted protein domains. The genomic sequence regions identified had varying nucleotide length ranging from 2187–4229 nucleotides (Table 4). Phvul.008G025200 with a coding length of 1266 nucleotides identified a region with homology on Pv04 that is different from the location identified by Phvul.008G022700. Phvul.008G025300 also had a single location on Pv03 with a high level of sequence similarity. As mentioned previously, the four annotated genes mentioned above did not identify any significant similarity with the other annotated genes found in this study. Six annotated genes within the group of 17 annotated genes that had a high level of sequence similarity to each other identified a short unique region with 333 nucleotides on Pv05 (Tables 2 and 4). The short length of this sequence on Pv05 is unlikely to be involved in a functional role. However, Gonzales et al. [32] recently reported the location of a main effect QTL with effects on leaf, stem and petiole for anthracnose resistance on Pv05. The other eleven annotated genes within the group of 17 did not identify any region with sequence similarity based on the stringency criteria used. Since this group is related to *COK–4* this further confirms the uniqueness of the *COK–4* locus and its genomic location in Pv08. The five annotated candidate genes that formed a second group with sequence similarity (Table 2) also identified a region on Pv04 with sequence similarity. Three of the five annotated genes, Phvul.008G029800, Phvul.008G030200 and Phvul.008G030400, identified a near identical region with few nucleotide differences on Pv04, whereas Phvul.008G030700 identified a region that was 62.88 kb away from the region identified by the other three members of the group (Table 4). The chromosome 4 region with homology identified by the fifth member of this group, Phvul.008G030800 was 16.3 kb away from the region identified by Phvul.008G030700. The annotated gene Phvul.008G031000 with sequence similarity to phosphoinositide-specific phospholipase C (PI-PLC) domain [20], also identified a region on Pv04 with sequence similarity. The PI-PLC domain also had some level of similarity with sequences on Pv01, Pv02, Pv03 and Pv11 but with lower stringency criteria than used in this study (data not shown). Phvul.008G031100 and Phvul.008G031200 did not identify any region with sequence similarity. The three annotated candidate genes that had predicted 1,3-β-glucanases activity, identified a distinct overlapping region on Pv04 (Table 4). It is noted that the chromosomes that had high level of sequence homology to the candidate genes found in the study such as Pv02, Pv03, Pv04 and Pv07 are known to have characterized major genes for anthracnose resistance [2].

**Table 4:** Results of the sequence homology searches between the 23 putative annotated candidate genes of Pv08 and the genomic sequences of G19833. A cutoff stringency E value of <E^−63^ was used in identifying genomic locations of similar sequences in the G19833 genome.

Annotated gene sequence	G19833 Genome	Target length	Score	E value	Coding length of the query
Phvul.008G021000	Chr03:2,112,207..2,114,774	2567	252	1.60E-64	2232
Phvul.008G022700*	Chr04:5,409,398..5,413,257	3859	482.8	4.50E-134	1872
Phvul.008G022700*	Chr03:5,041,232..5,045,064	3833	343.9	2.80E-92	1872
Phvul.008G022700*	Chr02:7,325,945..7,330,173	4229	273.6	4.20E-71	1872
Phvul.008G022700*	Chr07:8,620,038..8,622,224	2187	246.5	5.80E-63	1872
Phvul.008G025200	Chr04:4,990,688..4,992,970	2283	284.4	1.60E-74	1266
Phvul.008G025300	Chr03:3,770,715..3,772,559	1845	661.3	0	1926
Phvul.008G026600^a^	Chr05:20,356,072..20,356,404	333	448.5	5.00E-124	1038
Phvul.008G026700^a^	Chr05:20,356,072..20,356,404	333	601.8	2.30E-170	660
Phvul.008G026900^a^	Chr05:20,356,072..20,356,404	333	470.2	1.60E-130	1074
Phvul.008G027100^a^	Chr05:20,356,072..20,356,404	333	470.2	1.50E-130	1041
Phvul.008G027200^a^	Chr05:20,356,072..20,356,404	333	484.6	7.00E-135	1038
Phvul.008G027300^a^	Chr05:20,356,072..20,356,404	333	479.2	2.90E-133	1026
Phvul.008G028400^a^	None				897
Phvul.008G028200^a^	None				1035
Phvul.008G028300^a^	None				1047
Phvul.008G028500^a^	None				1044
Phvul.008G028600^a^	None				1128
Phvul.008G029500^a^	None				1032
Phvul.008G029600^a^	None				1014
Phvul.008G029700^a^	None				1101
Phvul.008G029800^b^	Chr04:4,330,558..4,331212	654	389	3.30E-106	828
Phvul.008G029900^a^	None				660
Phvul.008G030100^a^	None				1032
Phvul.008G030200^b^	Chr04:4,330,588..4,332,734	2147	585.6	7.10E-165	2595
Phvul.008G030400^b^	Chr04:4,330,567..4,332,733	2167	609	6.00E-172	2517
Phvul.008G030700^b^	Chr04:4,395,618..4,397,791	2174	580.2	3.00E-163	2577
Phvul.008G030800^b^	Chr04:4,414082..4,416416	2334	587.4	2.00E-165	2601
Phvul.008G031000	Chr04:4,365,204..4,366,960	1757	336.7	2.40E-90	1095
Phvul.008G031100	None				936
Phvul.008G031200	None				2784
Phvul.008G031300^a^	none				1020
Phvul.008G033000^c^	Chr04:5,761,767..5,768,973	7206	1294.3	0	2004
Phvul.008G033100^c^	Chr04:5,761404..5,769329	7925	11333	0	1968
Phvul.008G033200^c^	Chr04:5,761,768..5,768,973	7205	1105	0	1968

^a^17 annotated genes that form a cluster due to nucleotide/predicted protein similarity

^b^5 annotated genes that form a cluster due to nucleotide/predicted protein similarity

^c^3 annotated genes that form a cluster due to nucleotide/predicted protein similarity

*same annotated candidate gene that identified multiple locations with sequence similarity

## Conclusions

The *Co–4* resistance gene appears to be a complex locus containing the classical NBS-LRR and RLK type resistance genes in addition to PI-PLC genes and potential genes with β-glucanase activity. Although a majority of genes involved in conferring resistance against anthracnose seem to be *COK–4* related genes with functional domains containing LRR and kinase proteins, it is possible that there are other genes that are either unique or that exist in small clusters that are involved in resistance mechanisms. Further, the genes with potential β-glucanase activity may also play a role in conferring resistance against invading fungal pathogens. Although genes containing PI-PLC domains are not considered as classical genes with race specific resistance, it is possible that genes with PI-PLC domain activity also play a defense mechanism role in resistance conferred by *Co–4* as was suggested of the candidate genes present in the *Co-x* resistance gene. Since G19833 genotype does not possess known resistance to *C*. *lindemuthianum* at the Co–4 locus, the information generated from studying the sequence information can be used to more specifically characterize the nature and the organization of the *Co–4* locus by isolating and studying specific regions of the 936.5 kb region from a set of bean cultivars with varying levels of resistance to anthracnose.

## Supporting Information

S1 Fig Frequency distribution of anthracnose ratings of the 94 F_4-5_ recombinant inbred line population of B09197 x Nautica. (JPG)

S2 Fig Nucleotide alignment of 17 of the 23 annotated potential candidate genes that formed a group with each other: Phvul.008G026600, Phvul.008G026700, Phvul.008G026900, Phvul.008G027100, Phvul.008G027200, Phvul.008G027300, Phvul.008G028200, Phvul.008G028300, Phvul.008G028400, Phvul.008G028500, Phvul.008G028600, Phvul.008G029500, Phvul.008G029600, Phvul.008G029700, Phvul.008G029900, Phvul.008G030100 and Phvul.008G031300. (TIF)

S3 Fig Nucleotide alignment of the four annotated candidate genes Phvul.008G028300, Phvul.008G028400, Phvul.008G028500, Phvul.008G028600 with the COK–4 candidate gene AF153441. (TIF)

S4 Fig Amino acid alignment of the 5 annotated candidate genes that formed the second group with each other: Phvul.008G029800, Phvul.008G030200, Phvul.008G030400, Phvul.008G030700 and Phvul.008G030800. (JPG)

S5 Fig Amino acid alignment of the 3 annotated candidate genes that formed the third group with each other: Phvul.008G033000, Phvul.008G033100 and Phvul.008G033200 that had functional annotated domains of 1,3 –β-glucanase. (JPG)

S1 File Genotypic and Phenotypic data collected on members of the recombinant inbred line population, SNP Marker physical location, and the outcome of the one-way ANOVA in single marker QTL analysis for the SNP Markers. (XLSX)

S1 Table Annotated genes, size of the genomic sequence and the predicted functional annotation. (PDF)
