# Supplementary figures and images for: Candidate Gene Identification with SNP Marker-Based Fine Mapping of Anthracnose Resistance Gene Co-4 in Common Bean
Source: PLoS One. 2015 Oct 2;10(10):e0139450. doi: 10.1371/journal.pone.0139450 (PMC4592015; doi:10.1371/journal.pone.0139450)

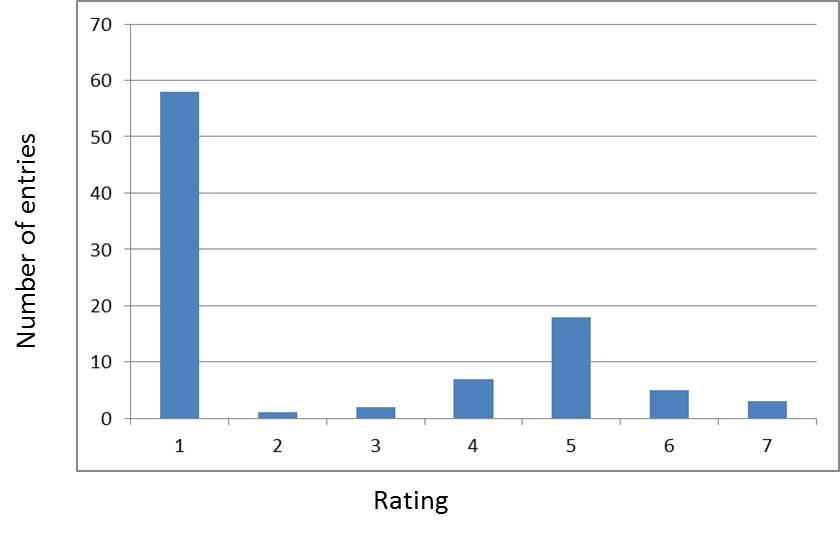

Supplement: S1 Fig — (JPG) [file pone.0139450.s001.jpg]

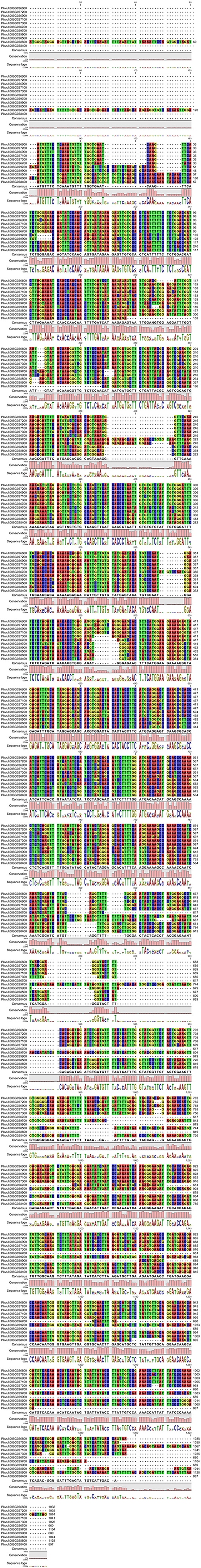

Supplement: S2 Fig — (TIF) [file pone.0139450.s002.tif]

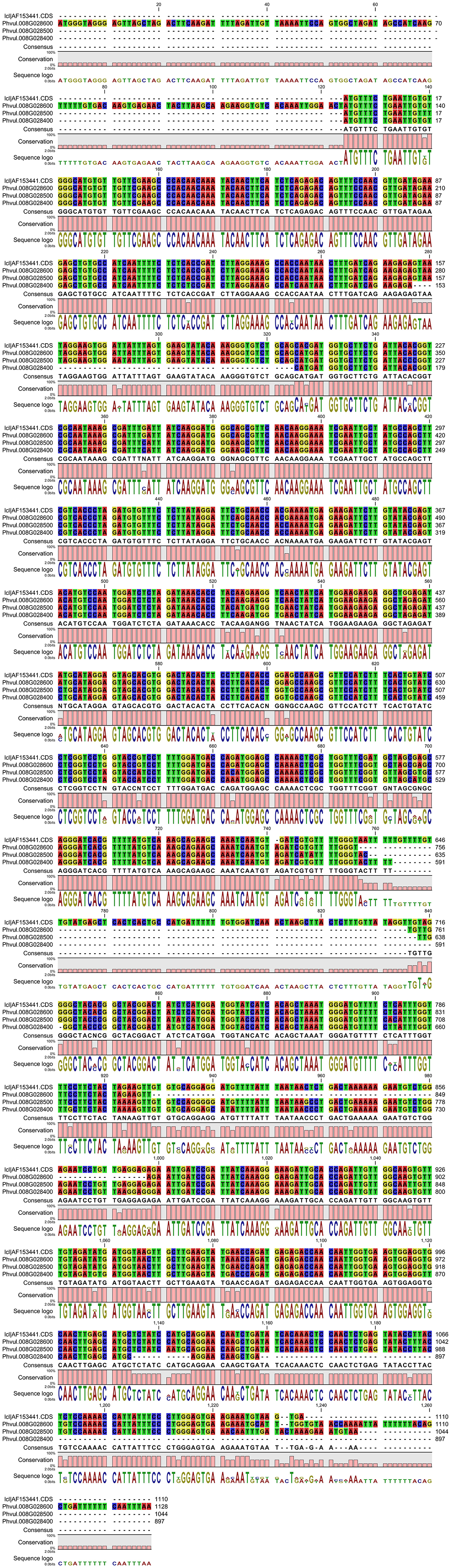

Supplement: S3 Fig — (TIF) [file pone.0139450.s003.tif]

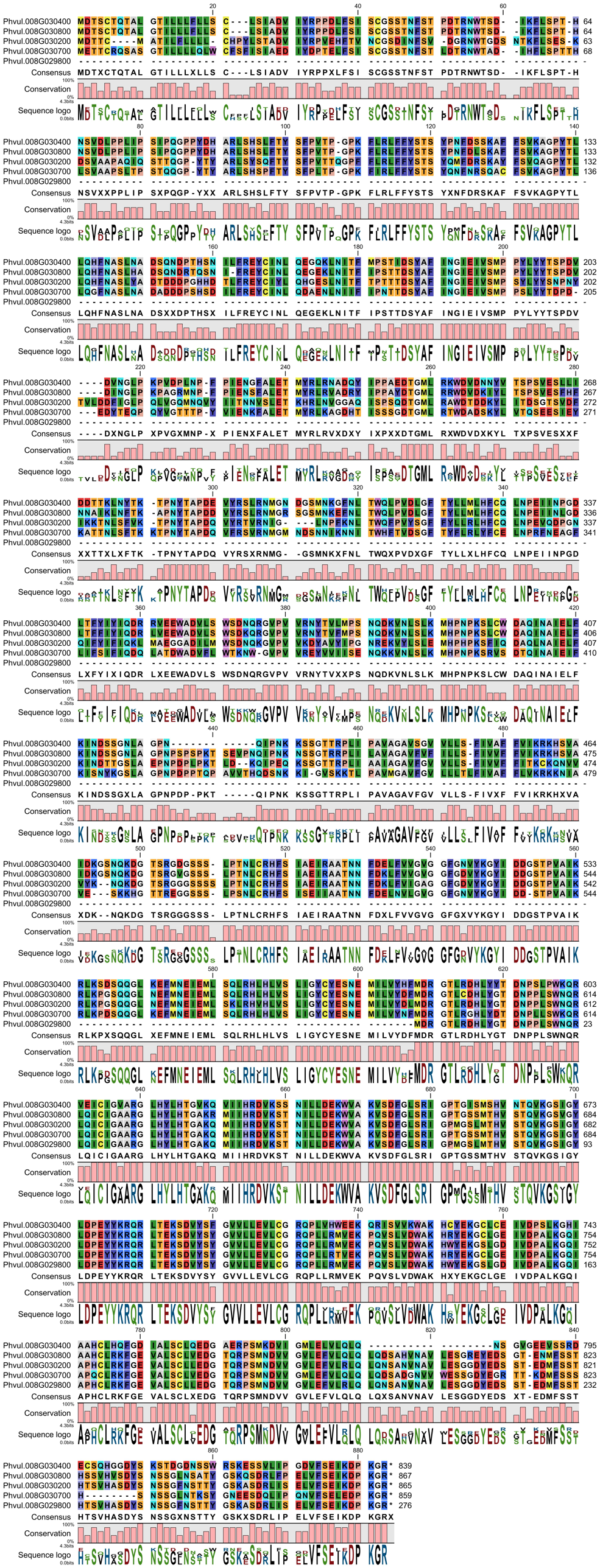

Supplement: S4 Fig — (JPG) [file pone.0139450.s004.jpg]

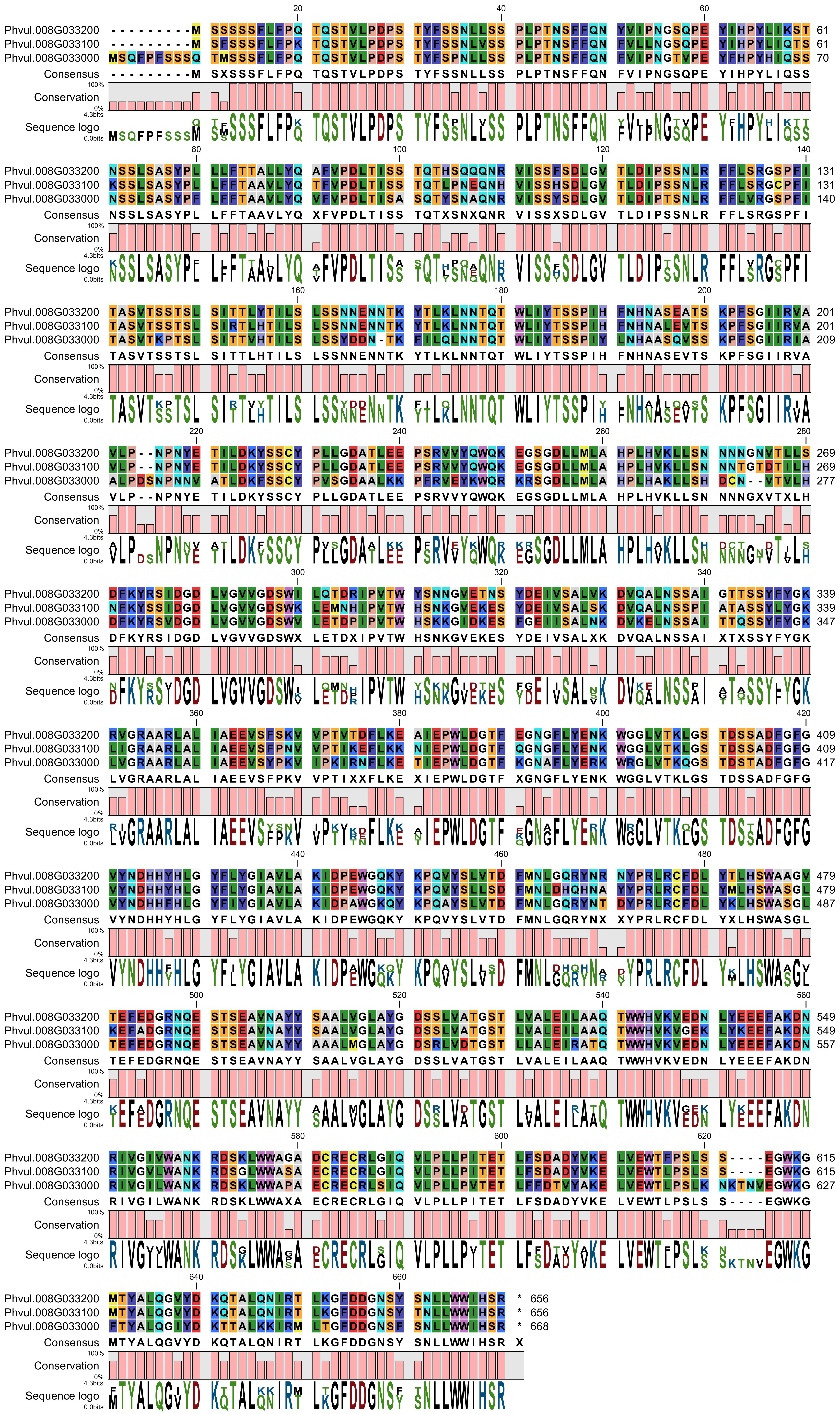

Supplement: S5 Fig — (JPG) [file pone.0139450.s005.jpg]
