# Supplementary material for: Candidate Gene Identification with SNP Marker-Based Fine Mapping of Anthracnose Resistance Gene Co-4 in Common Bean
Source: PLoS One. 2015 Oct 2;10(10):e0139450. doi: 10.1371/journal.pone.0139450 (PMC4592015; doi:10.1371/journal.pone.0139450)
Supplement: S1 Table — (PDF) [file pone.0139450.s007.pdf]

Supplementary Table S1

**Annotated Gene**	**Genomic Sequence Length**	**Predicted functional annotation**
Phvul.008G004500	3688	Chaparonin containing T-complex protein
Phvul.008G004600	1863	Carboxylesterase related
Phvul.008G004700	35676	Uncharactrized protein
Phvul.008G004800	5740	Flavodoxin related
Phvul.008G004900	2132	Uncharactrized protein
Phvul.008G005000	1845	Uncharactrized protein
Phvul.008G005100	935	Ribosomal protein
Phvul.008G005200	2068	Uncharactrized protein
Phvul.008G005300	1696	Cytokinin synthase
Phvul.008G005400	2224	Ribosomal protein
Phvul.008G005500	1527	Uncharactrized protein
Phvul.008G005600	4380	Cytochrome P450
Phvul.008G005700	3606	Transporter family
Phvul.008G005800	2340	Uncharactrized protein
Phvul.008G005900	3253	Uncharactrized protein
Phvul.008G006000	1126	Uncharactrized protein
Phvul.008G006100	3478	Chloride Peroxidase
Phvul.008G006200	4346	Mannose-6-phosphate isomerase
Phvul.008G006300	1002	Uncharactrized protein
Phvul.008G006400	9321	Uncharactrized protein
Phvul.008G006500	6328	U1 small nuclear ribonucleoprotein
Phvul.008G006600	1674	Uncharactrized protein
Phvul.008G006700	3661	rRNA processing protein RRP7
Phvul.008G006800	6629	Ribosome Assembly protein
Phvul.008G006900	1595	Uncharactrized protein
Phvul.008G007000	268	Uncharactrized protein
Phvul.008G007100	4968	Phosphatidylinositol N-acetylglucosaminyl transferase
Phvul.008G007200	926	Uncharactrized protein
Phvul.008G007300	4066	Peptidyl-tRNA hydrolase
Phvul.008G007400	1338	Uncharactrized protein
Phvul.008G007500	6254	transmembrane transport
Phvul.008G007600	1995	Multitransmembrane protein
Phvul.008G007700	6380	RNA binding
Phvul.008G007800	435	Uncharactrized protein
Phvul.008G007900	7911	Hypothetical protein
Phvul.008G008000	774	Uncharactrized protein
Phvul.008G008100	2177	hydrolase activity
Phvul.008G008200	861	Protease inhibitor/seed storage

Phvul.008G008300	9036	protein binding
Phvul.008G008400	2560	Uncharactrized protein
Phvul.008G008500	4007	catalytic activity
Phvul.008G008600	393	Uncharactrized protein
Phvul.008G008700	402	Uncharactrized protein
Phvul.008G008800	595	Uncharactrized protein
Phvul.008G008900	3059	Histone like transcription factor
Phvul.008G009000	4476	Uncharacterized membrane protein, predicted efflux pump
Phvul.008G009100	3320	Uncharactrized protein
Phvul.008G009200	13477	ATP-dependent DNA ligase
Phvul.008G009300	4488	protein methyltransferase activity
Phvul.008G009400	4043	protein folding
Phvul.008G009500	692	Uncharactrized protein
Phvul.008G009600	2641	Translation initiation factor 5 (eIF-5)
Phvul.008G009700	3151	Translation initiation factor 5 (eIF-5)
Phvul.008G009800	2038	Membrane associated ring finger
Phvul.008G009900	2717	Uncharactrized protein
Phvul.008G010000	3002	ATP-dependent Clp protease, proteolytic subunit
Phvul.008G010100	7158	Phenylalanyl-tRNA synthetase
Phvul.008G010200	1640	Copper chaperone
Phvul.008G010300	2401	Uncharactrized protein
Phvul.008G010400	804	Uncharactrized protein
Phvul.008G010500	393	Uncharactrized protein
Phvul.008G010600	2256	hydrolase activity, hydrolyzing O-glycosyl compounds
Phvul.008G010700	2339	Uncharactrized protein
Phvul.008G010800	5230	Glucosyl transferase
Phvul.008G010900	2885	phospholipid binding
Phvul.008G011000	3622	Actin and related proteins
Phvul.008G011100	841	Uncharactrized protein
Phvul.008G011200	2999	Uncharactrized protein
Phvul.008G011300	1910	ATP binding
Phvul.008G011400	2719	ATP binding
Phvul.008G011500	723	protein-disulfide reductase activity
Phvul.008G011600	2706	ATP binding
Phvul.008G011700	797	Transmembrane transport
Phvul.008G011800	1587	protein-disulfide reductase activity
Phvul.008G011900	2705	ATP binding
Phvul.008G012000	584	Uncharactrized protein
Phvul.008G012100	7942	transcription initiation factor TFIID subunit 6
Phvul.008G012200	1553	Uncharactrized protein
Phvul.008G012300	4261	Uncharactrized protein
Phvul.008G012400	4265	RNA binding
Phvul.008G012500	1813	DNA binding

Phvul.008G012600	2930	ATP binding
Phvul.008G012700	4192	Uncharactrized protein
Phvul.008G012800	5269	Protein geranylgeranyltransferase Type I, beta subunit
Phvul.008G012900	2015	Uncharactrized protein
Phvul.008G013000	2996	ATP binding
Phvul.008G013100	4158	Uncharactrized protein
Phvul.008G013200	918	catalytic activity
Phvul.008G013300	3454	Subtilisin-like proptease
Phvul.008G013400	8299	Phospholipase D
Phvul.008G013500	18529	Uncharactrized protein
Phvul.008G013600	2606	RNA and export factor binding protein
Phvul.008G013700	9905	Putative zinc-finger domain
Phvul.008G013800	855	DNA repair protein RHP57
Phvul.008G013900	2646	Uncharactrized protein
Phvul.008G014000	5278	regulation of transcription, DNA-dependent
Phvul.008G014100	255	Uncharactrized protein
Phvul.008G014200	4620	Uncharactrized protein
Phvul.008G014300	4420	Ras-related protein Rab-8A
Phvul.008G014400	6977	2-Oxoglutarate dehydrogenase
Phvul.008G014500	6055	Uncharactrized protein
Phvul.008G014600	633	Uncharactrized protein
Phvul.008G014700	3040	Uncharacterized protein
Phvul.008G014800	2170	F-box domain
Phvul.008G014900	2054	Aquaporin Transporter
Phvul.008G015000	9610	Peptide exporter, ABC superfamily
Phvul.008G015100	2443	Hypothetical protein
Phvul.008G015200	1651	large subunit ribosomal protein
Phvul.008G015300	5185	Hypothetical protein
Phvul.008G015400	8085	Beta-N-acetylhexosaminidase
Phvul.008G015500	4168	Cation Efflux Protein/ Zinc Transporter
Phvul.008G015600	8282	Sulfate Transporter
Phvul.008G015700	2441	Hypothetical protein
Phvul.008G015800	2184	Hypothetical protein
Phvul.008G015900	1711	Plant protein 1589 of unknown function
Phvul.008G016000	4563	Uncharacterized protein
Phvul.008G016100	2497	Hypothetical protein
Phvul.008G016200	609	Uncharacterized protein
Phvul.008G016300	404	Short sequence
Phvul.008G016400	402	Short sequence
Phvul.008G016500	1233	IQ calmodulin-binding motif
Phvul.008G016600	1274	IQ calmodulin-binding motif
Phvul.008G016700	4544	Amidohydrolase
Phvul.008G016800	3012	TRNA-nucleotidyl transferase/poly(A) polymerase family member

Phvul.008G016900	4692	Glucose-6-phosphate dehydrogenase
Phvul.008G017000	2957	OTU-like cysteine protease
Phvul.008G017100	2733	Uncharacterized protein
Phvul.008G017200	6392	Sphinganine-1-phosphate aldolase
Phvul.008G017300	4363	Eukaryotic translation initiation factor 3 related
Phvul.008G017400	3815	Uncharacterized protein
Phvul.008G017500	5256	G-protein beta subunit-like protein
Phvul.008G017600	8122	Uncharacterized conserved protein
Phvul.008G017700	6493	Protein of unknown function
Phvul.008G017800	4608	Aldo/Keto reductase
Phvul.008G017900	1578	Exotosin (Heparan sulfate glycosyl transferase) - related
Phvul.008G018000	1794	P21-Rho-binding domain
Phvul.008G018100	3548	Chitinase related
Phvul.008G018200	5065	Protein phosphatase 2C delta
Phvul.008G018300	11097	Chloroplast nucleiod DNA binding related
Phvul.008G018400	15651	RNA-binding protein 7,8
Phvul.008G018500	12514	PHD Finger protein
Phvul.008G018600	336	Protein binding domain
Phvul.008G018700	1707	Nucleoporin related
Phvul.008G018800	8723	Ring finger protein
Phvul.008G018900	1193	Uncharacterized protein
Phvul.008G019000	6507	RNA polymerase
Phvul.008G019100	3579	Serine carboxypeptidase II
Phvul.008G019200	2925	Hypothetical protein
Phvul.008G019300	9468	GCN-4 Complementing protein
Phvul.008G019400	4047	Uncharacterized protein
Phvul.008G019500	8292	Protein Mei 2, essential for commitment to meiosis, and related proteins
Phvul.008G019600	4754	Hypothetical protein
Phvul.008G019700	3049	N-Terminal Acetyl transferase
Phvul.008G019800	1642	Ribosomal protein L30
Phvul.008G019900	1113	Hypothetical protein
Phvul.008G020000	2359	DNA-Directed RNA Polymerase
Phvul.008G020100	3561	Uncharacterized protein
Phvul.008G020200	2030	Allene oxide cyclase
Phvul.008G020300	1287	Uncharactrized protein
Phvul.008G020400	2066	TCP family transcription factor
Phvul.008G020500	5877	Translation initiation factor eIF-2B subunit
Phvul.008G020600	6888	Transcription factor
Phvul.008G020700	4330	Hypothetical Protein
Phvul.008G020800	2503	Oligopeptide Transporter related
Phvul.008G020900	1376	NB-ARC domain
Phvul.008G021100	2187	Hypothetical protein
Phvul.008G021200	5075	Uncharactrized protein

Phvul.008G021300	659	Uncharactrized protein
Phvul.008G021400	6624	Hypothetical protein
Phvul.008G021500	9993	Tetratricopeptide repeat
Phvul.008G021600	4658	Uncharactrized protein
Phvul.008G021700	2573	RNA POLYMERASE III (DNA DIRECTED), 39KD SUBUNIT-RELATED
Phvul.008G021800	804	Hypothetical protein
Phvul.008G021900	3276	protein YIF1B-like
Phvul.008G022000	3006	proline dehydrogenase 2
Phvul.008G022100	2557	S-adenosylmethionine decarboxylase
Phvul.008G022200	234	short sequence (153 bp)
Phvul.008G022300	1162	Short sequence (192 nt)
Phvul.008G022400	1066	short seq (228 nt) - zinc finger protein
Phvul.008G022500	1209	short-chain dehydrogenase reductase 2a-like
Phvul.008G022600	2248	pentatricopeptide repeat-containing protein
Phvul.008G022800	2687	Zinc finger protein
Phvul.008G022900	2246	Uncharactrized protein/Ribosomal protein
Phvul.008G023000	2420	Ribosomal or hypothetical proteins
Phvul.008G023100	502	short seq (255 nt) - self-incompatibility response (SCRL) protein
Phvul.008G023200	623	short sequence (255)
Phvul.008G023300	513	short sequence
Phvul.008G023400	918	Uncharactrized protein
Phvul.008G023500	782	Uncharactrized protein
Phvul.008G023600	449	Uncharactrized protein
Phvul.008G023700	2768	6-phosphogluconate dehydrogenase (GND), transcript variant
Phvul.008G023800	864	bZIP transcription factor
Phvul.008G023900	3530	pentatricopeptide repeat-containing protein
Phvul.008G024000	4999	WPP domain-interacting tail-anchored protein
Phvul.008G024100	3594	sodium-coupled neutral amino acid transporter 1
Phvul.008G024200	1680	Uncharacterized protein
Phvul.008G024300	3496	pentatricopeptide repeat-containing protein
Phvul.008G024400	3959	pentatricopeptide repeat-containing protein
Phvul.008G024500	8093	Glycine max kanadaptin-like (LOC100778453), transcript variant X2, misc_RNA
Phvul.008G024600	12414	Glycine max DNA-directed RNA polymerase 2B, chloroplastic/mitochondrial-like
Phvul.008G024700	1201	Hypothetical protein
Phvul.008G024800	3756	Uncharactrized protein
Phvul.008G024900	1443	Hypothetical protein
Phvul.008G025000	2426	F-box/kelch-repeat protein
Phvul.008G025100	1788	Uncharactrized protein
Phvul.008G025400	5399	Uncharactrized protein
Phvul.008G025500	3681	Glycine max transcription factor BIM1-like (LOC100791730), mRNA
Phvul.008G025600	3525	Uncharacterized protein/hypothetical protein

Phvul.008G025700	21182	Auxin transporter protein?? Zinc finger type protein
Phvul.008G025800	4018	Hypothetical protein
Phvul.008G025900	2431	pentatricopeptide repeat-containing protein
Phvul.008G026000	1971	Hypothetical protein
Phvul.008G026100	4102	UDP-glycosyltransferase 73C3-like
Phvul.008G026200	1727	Uncharactrized protein
Phvul.008G026300	1435	Glycine max proline-rich extensin-like protein EPR1-like mRNA
Phvul.008G026400	9074	Hypothetical / uncharacterized proteins
Phvul.008G026500	4733	Glycine max E3 ubiquitin-protein ligase
Phvul.008G026800	1953	Hypothetical protein
Phvul.008G027000	3109	Uncharactrized protein
Phvul.008G027400	759	Hypothetical protein
Phvul.008G027500	6363	ubiquitin-protein ligase
Phvul.008G027600	2148	Hypothetical protein
Phvul.008G027700	5181	Glycine max probable E3 ubiquitin-protein ligase HERC2-like transcript variant
Phvul.008G027800	7918	MADS-box protein
Phvul.008G027900	6061	Developmental potein
Phvul.008G028000	1734	transcription factor MYB59-like
Phvul.008G028100	2760	Hypothetical protein
Phvul.008G028700	986	Uncharactrized protein
Phvul.008G028800	4357	Cytochrome Proteins
Phvul.008G028900	2448	alpha 1,4 glucan protein synthase
Phvul.008G029000	2368	Glycine max COBRA-like protein 4-like transcript variant X1, misc_RNA
Phvul.008G029100	3704	protein COBRA-like
Phvul.008G029200	3521	protein COBRA-like
Phvul.008G029300	5004	Predicted mitochondrial carrier protein
Phvul.008G029400	1630	Uncharactrized protein
Phvul.008G030000	1127	Hypothetical protein
Phvul.008G030300	655	Hypothetical protein
Phvul.008G030500	2702	Hypothetical protein
Phvul.008G030600	1146	xylosyltransferase 2-like/B-1-3-galactosyl-o-glycosyl-glycoprotein
Phvul.008G030900	683	Hypothetical protein
Phvul.008G031400	5771	ALPHA/BETA HYDROLASE FOLD-CONTAINING PROTEIN
Phvul.008G031500	5463	Glycine max ubiquitin-like domain-containing CTD phosphatase 1-like
Phvul.008G031600	8146	Uncharactrized protein
Phvul.008G031700	2173	Hypothetical protein
Phvul.008G031800	1663	Calmodulin binding proteins
Phvul.008G031900	1473	phenolic glucoside malonyltransferase 2
Phvul.008G032000	2052	phenolic glucoside malonyltransferase
Phvul.008G032100	1676	Glycine max phenolic glucoside malonyltransferase 1-like mRNA
Phvul.008G032200	1685	phenolic glucoside malonyltransferase 1-like
Phvul.008G032300	1567	Hypothetical protein

Phvul.008G032400	1452	Glycine max phenolic glucoside malonyltransferase 1-like mRNA
Phvul.008G032500	1217	Hypothetical protein
Phvul.008G032600	2652	Glycine max isoflavone 7-O-methyltransferase-like
Phvul.008G032700	1544	isoflavone 7-O-methyltransferase-like
Phvul.008G032800	10203	ras GTPase-activating protein-binding protein 2-like transcript variant mRNA
Phvul.008G032900	3337	PREDICTED: Glycine max ninja-family protein AFP2-like mRNA
Phvul.008G033300	5609	Glycine max serine carboxypeptidase-like 25-like transcript variant mRNA
Phvul.008G033400	2735	Exocyst complex component
Phvul.008G033500	3079	Glycine rich RNA binding proteins
Phvul.008G033600	9750	protein RIK-like (LOC100806616), transcript variant
Phvul.008G033700	971	Histone H3
Phvul.008G033800	11298	Uncharacterized protein
Phvul.008G033900	2198	Hypothetical protein/RNA splicing protein
Phvul.008G034000	1108	Glycosyltransferase
